# Juvenile bovine bone is an appropriate surrogate for normal and reduced density human bone in biomechanical testing: a validation study

**DOI:** 10.1038/s41598-018-28155-w

**Published:** 2018-07-05

**Authors:** J. W. A. Fletcher, S. Williams, M. R. Whitehouse, H. S. Gill, E. Preatoni

**Affiliations:** 1 Department for Health, University of Bath, Bath, UK; 2 Musculoskeletal Research Unit, Bristol Medical School, University of Bristol, Bristol, UK; 3 Department of Mechanical Engineering, University of Bath, Bath, UK

## Abstract

Orthopaedic research necessitates accurate and reliable models of human bone to enable biomechanical discoveries and translation into clinical scenarios. Juvenile bovine bone is postulated to be a potential model of normal human bone given its dimensions and comparatively reduced ethical restrictions. Demineralisation techniques can reduce bone density and alter bone properties, and methods to model osteoporotic bone using demineralised juvenile bovine bone are investigated. Juvenile bovine long bones were quantitatively CT scanned to assess bone density. Demineralisation using hydrochloric acid (0.6, 1.2 and 2.4 M) was performed to create different bone density models which underwent biomechanical validation for normal and osteoporotic bone models. All long bones were found to have comparable features to normal human bone including bone density (1.96 ± 0.08 gcm^−3^), screw insertion torque and pullout strength. Demineralisation significantly reduced bone density and pullout strength for all types, with 0.6 M hydrochloric acid creating reductions of 25% and 71% respectively. Juvenile bovine bone is inexpensive, easy to source and not subject to extensive ethical procedures. This study establishes for the first time, the use of its long bones as surrogates for both normal and osteoporotic human specimens and offers preliminary validation for its use in biomechanical testing.

## Introduction

Orthopaedic research necessitates access to accurate and reliable models of human bone to enable biomechanical and clinical advancements. A significant clinical driver for orthopaedic research is the increasing rate of fractures, particularly in patients with osteoporotic bone. Given the high failure rate of fracture fixations^1,2^ improvements are urgently needed for which the underlying research requires reliable and readily available specimens. Thus, the impact from advancements in available experimental bone models is becoming ever greater. Various cadaveric and *in vivo* animal models are available for experimental testing of methods and screw designs for fixing fractures, with the option available to alter their material properties through demineralisation using chemical treatments. However, all models are associated with limitations. Cadaveric human bone often only represents an older demographic of the population and the interspecimen variability^3,4^ means that large numbers are needed to appropriately power studies. There are also ethical constraints associated with the procurement, storage, usage and disposal of human specimens. Models using *in vivo* modification of animal bone have been established, such as ovariectomised animals, but these can fail to produce the desired bone properties^5,6^, such as only mildly reducing bone density, whilst having macroscopic dimensions that are unrepresentative of human bone.

Unmodified *in vitro* animal models may have baseline properties incomparable to human bone, such as a higher volumetric bone mineral density (vBMD) and thicker cortices. Some of these characteristics can be modified with chemical treatments, such as hydrochloric acid (HCl), to demineralise bone^7–10^. However the macroscopic dimensions such as length and diameter cannot be easily changed. Despite 55–80% of fractures involving long bones^11,12^, no *in vitro* model using demineralisation techniques (or variants thereof) has been used on long bones; these methods have only been employed in instances using spinal vertebrae^7–10^.

Bovine bone has been used for modelling normal and osteoporotic bone, and has been shown to have high reliability^8^. However, the macroscopic properties of mature bovine long bones reduce their modelling accuracy as they are longer, with much thicker cortices than human bone. This limits the validity and the transferability of any biomechanical results to human *in vivo* clinical applications. Following the observation that juvenile bovine bone has comparable macroscopic dimensions to adult human bone, further investigation into the use of this as a model was postulated. Juvenile bovine bone has neither been investigated for its potential to biomechanically mimic human long bone, nor as a potential model of osteoporosis once demineralised. If the model is shown to be valid, this will offer a substantial change to testing practice as it represents an economical and viable alternative to the more expensive methods used currently. Also, it will offer a controllable way of reproducing the spectrum of densities seen in human samples; human bone characteristics are variable, but in biomechanical testing variables need to be controlled, and validated demineralisation techniques potentially offer this.

Our objectives were to establish and validate juvenile bovine bone as an appropriate model for biomechanical testing. Firstly, we established whether the vBMD of juvenile bovine long bones is comparable to literature quoted values for adult human bone. Secondly, we assessed the effect of acid demineralisation on vBMD, aiming to reduce this to replicate osteoporotic bone. Using different modification and preparation techniques, our tertiary objective was to analyse one specific type of long bone in detail (this bone being chosen based on its vBMD and ease of use) to assess if the modification techniques, including dehydration of samples, would reliably reduce the vBMD to create a spectrum of osteoporotic bone models. Our final objective was to validate the models using pull-out testing; this being the key requirement of a model being used for fracture fixation experiments.

## Methods

**Figure 1:**
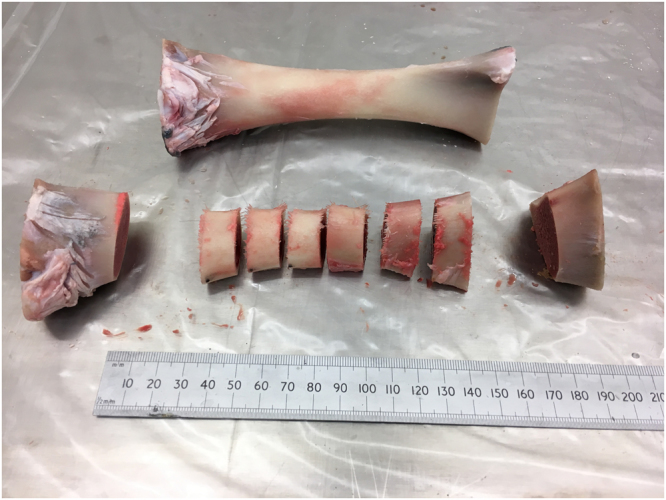
Sectioning of long bone diaphysis, with six cut diaphyseal samples retained for testing.

**Figure 2:**
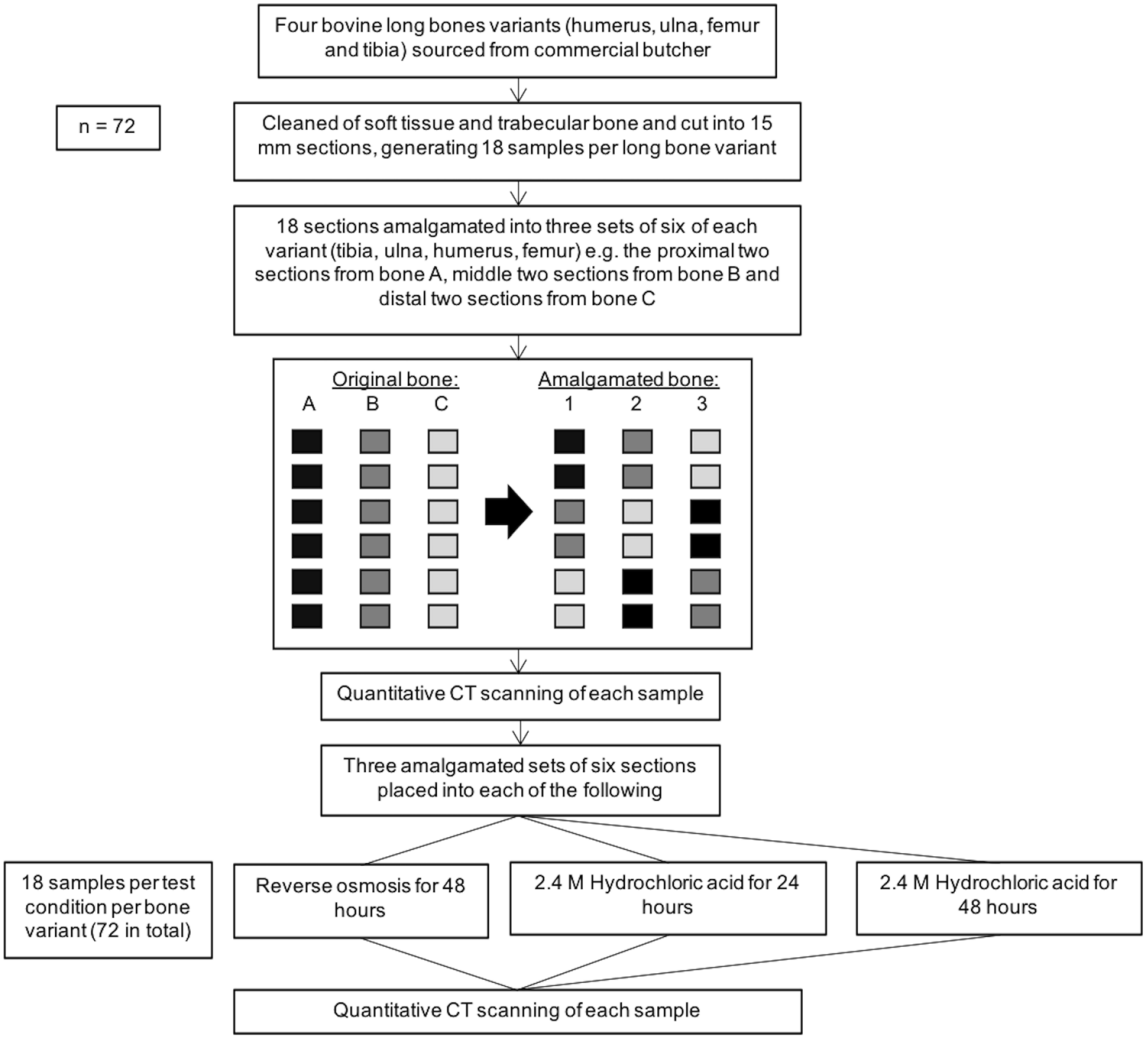
Preparation of long bone variants for bone mineral density measurements.

Four variants of long bones (humerus, ulna, femur and tibia) from 4 to 5 month old calves were obtained from a commercial butcher (Bartlett and Sons, Bath, UK). All soft tissues, residual trabecular bone and metaphyseal areas were removed, before the remaining cortical diaphyses were sectioned using a circular saw into 15 mm length cross sections (Fig. 1); generating six samples per bone. The diaphyseal portion of the long bones was selected due to its more cylindrical shape and ease of use. Three specimens of each of the four long bone variants were used, each cut into six sections, generating 18 samples for testing under three conditions, detailed below (n = 72). Bone sets were amalgamated from the three different bones of each variety so that any variation in bone density between samples would be negated (Fig. 2). Based on preliminary data, non-inferiority power calculations showed that 12 samples would be needed to be 90% sure that the lower limit of a 90% two-sided confidence interval would be found, at a non-inferiority limit of 0.10 gcm^−3^. Each sample was clamped and 2.5 mm pilot holes were perpendicularly drilled using a bench drill, with the holes spaced equally around the circumference, at least 8 mm apart, with no more than six holes per sample.

Initially, assessment of volumetric bone mineral density of the four long bone variants was achieved by performing quantitative micro-CT analysis (X Tec, XT H 225 ST, Nikon Metrology UK Ltd, Derby, UK) of all samples before treatment; scanning protocols were the same for all samples (162 kV, resolution 0.2 mm) (Fig. 2). To assess the effect of demineralisation, 12 sets containing six samples were randomly selected (n = 72), weighed and placed in the three following solutions: reverse osmosis (R/O) water for 48 hours, 2.4 M HCl for 24 hours and 2.4 M HCl for 48 hours. For the 48 hours treatments, the solutions were changed at 24 hours. Each set was placed in a container of 1.5 l of solution to ensure that there would be an excess of demineralisation solution (>21 cm^3^ of HCl per 1 g of bone)^10^.

Samples were placed in a fume cupboard at 21 °C for the desired time period. They were thoroughly washed with running water following treatment, until a neutral pH was achieved, followed by repeat micro-CT scanning. Analysis of the CT data was performed using Simpleware ScanIP (Synopsys, Inc., Exeter, UK (release version 2017)). Phantoms of known density were used as controls, allowing for calibration of the CT grayscales to vBMD using linear regression.

**Figure 3:**
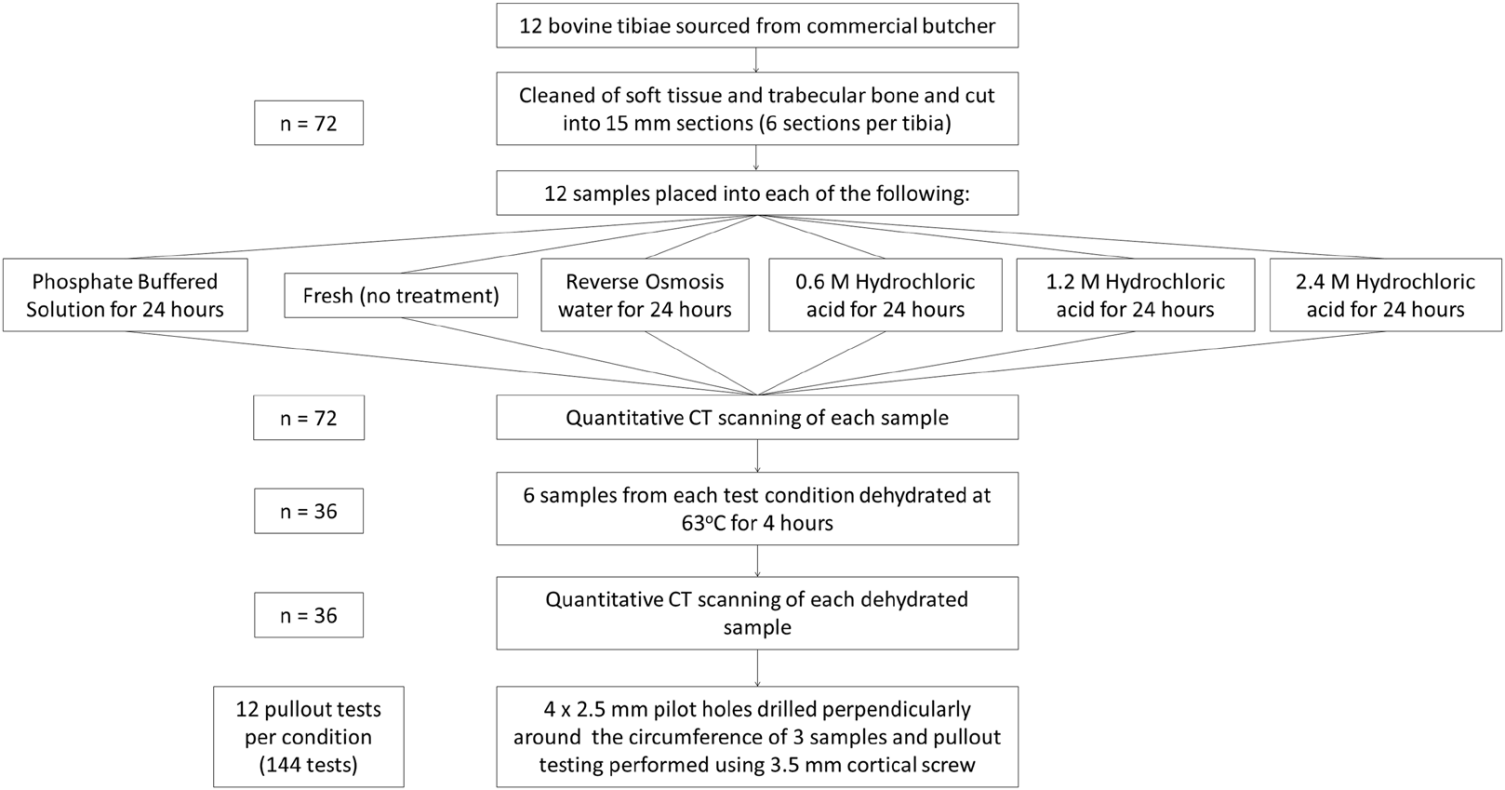
Preparation and testing of samples for different preparation and demineralisation techniques.

Following quantitative analysis of the four variants’ dimensions and vBMD, further testing of preparation and demineralisation techniques was performed on tibial samples, due to their long, straight diaphyseal portion. Twelve tibiae were prepared as before (giving n = 72 test specimens) (Fig. 3). Samples were tested under each of the six following conditions: fresh (tested within six hours of slaughter), R/O for 24 hours, phosphate buffered solution (PBS) (Sigma Aldrich Co. Ltd., Irvine, UK) for 24 hours, 0.6 M HCl for 24 hours, 1.2 M HCl for 24 hours or 2.4 M HCl for 24 hours. To assess the impact of drying of the samples, half of them (n = 36) were tested under the same six conditions but were dried for four hours at 63 °C following removal from their solution; generating twelve test conditions for the tibial samples. Again, quantitative micro-CT analysis was performed pre and post treatment.

### Pullout testing

Following CT scanning, samples underwent biomechanical testing. Small fragment cortical trauma screws (3.5 mm diameter, 18 mm length, (Stryker, Newbury, UK)) were manually inserted into the predrilled holes by the same, experienced orthopaedic surgeon mimicking clinical insertion methods (n = 144, 12 per test condition). The insertion torque was continuously recorded using a digital torque screwdriver (Torqueleader, MHH Engineering co. Ltd., Guildford, UK) to ensure that the theoretical maximum insertion torque was not exceeded. The stripping torque was predicted using theoretical equations for the maximum which were adjusted based on the observed material properties of pilot samples^13^. Screws were tightened to 0.5 Nm for all except demineralised samples where a stopping torque of 50% of the stripping torque was chosen, to ensure that the stopping torque was below the stripping torque.

**Figure 4:**
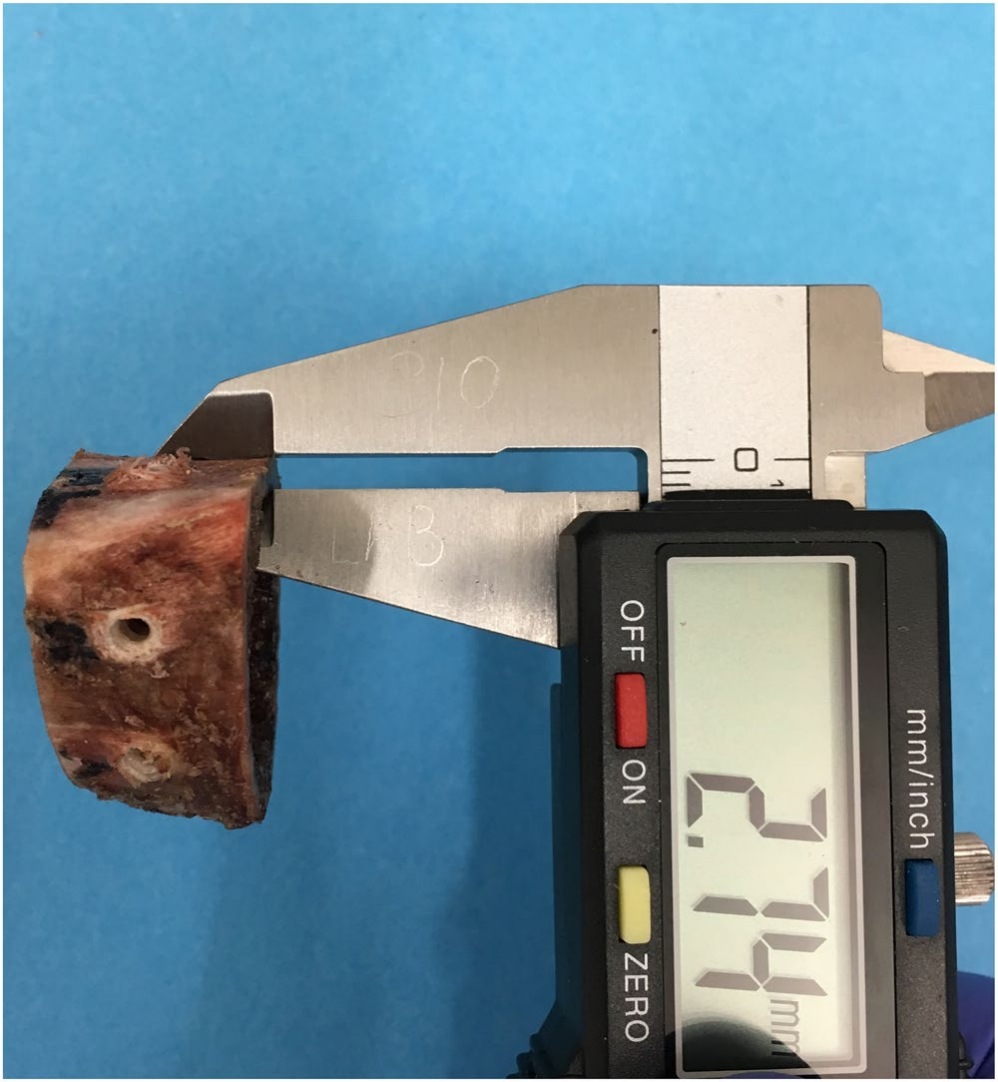
Measuring cortical thickness of juvenile bovine sample.

**Figure 5:**
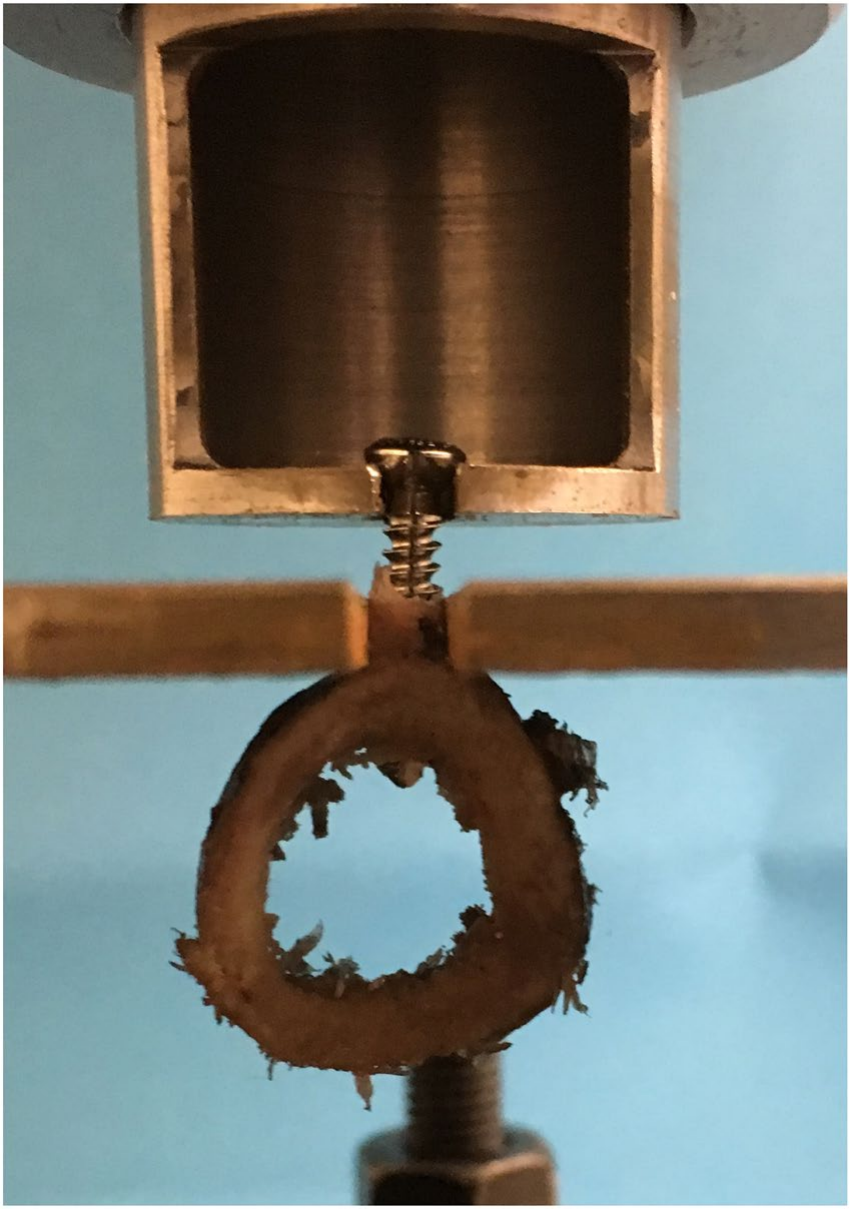
Mounted sample under testing in custom made jigs.

Cortical thickness, which dictates the number of screw threads engaged within the bone, correlates with pullout strength^14^, thus the relationship between cortical thickness and pullout strength was established using linear regression analysis. The cortices were measured using digital callipers (Fig. 4), assessing both proximal and distal aspects of the sample, with the mean value being used. The relationship between cortical thickness and pullout force was recorded for each testing condition so that linear regression analysis could be used to adjust the raw values to the mean cortical thickness. Samples were restrained with custom made jigs (Fig. 5). Twelve axial pullout tests (Instron, High Wycombe, UK) were performed per sample immediately after screw insertion, distracting at a strain rate seen in physiological conditions^15^ of 5 mm/min, recording at 20 Hz until the maximum force was demonstrated (using Bluehill software (Bluehill, Instron, High Wycombe, UK)).

Statistical analyses were performed using IBM SPSS (Version 22, IBM, New York, USA), with significance accepted at *p* ≤ 0.05. A one-way independent analysis of variance test (ANOVA) was used to examine differences in density between fresh bone types (humerus, ulna, femur and tibia). A two-way independent ANOVA was used to explore interactions in density between preparation types (R/O water for 48 hours, 2.4 M HCL for 24 hours and 2.4 M HCL for 48 hour) and bone types. Finally, a one-way independent ANOVA was used to examine differences in pull-out force between bone types. In cases where multiple comparisons were made within a given variable, a Bonferroni adjustment was made to prevent inflation of Type I error rate. The raw data is available at the following online repository: [https://doi.org/10.15125/BATH-00410]^16^.

## Results

### Comparison of different long bones and response to demineralisation

The initial analysis of the long bones showed no difference in mean vBMD between all four types (Table 1). Demineralising samples in 2.4 M HCl for 24 hours and 48 hours produced reductions in mean vBMD of 39% (p < 0.001) and 41% (p < 0.001) respectively. Whilst treatment for 48 hours reduced the densities the most, post-hoc ANOVA showed this was not significantly more than the reduction at 24 hours (p = 0.159). There were no significant differences in the percentage reduction in vBMD between the different long bones.

**Table 1:** Volumetric bone mineral density (vBMD) of four long bones and percentage reductions compared to fresh tibia.

Bone Variant	Standard sample vBMD (gcm^−3^)	48 hr R/O vBMD (gcm^−3^)	24 hours 2.4 M HCl		48 hours 2.4 M HCl	
			vBMD (gcm^−3^)	% reduction from normal within each bone variant	vBMD (gcm^−3^)	% reduction from normal within each bone variant
Tibia	1.93 ± 0.08	2.01 ± 0.02	1.20 ± 0.04	38	1.16 ± 0.03	40
Femur	1.98 ± 0.09	1.99 ± 0.06	1.19 ± 0.08	40	1.18 ± 0.03	40
Humerus	1.96 ± 0.08	2.07 ± 0.04	1.18 ± 0.02	40	1.15 ± 0.01	41
Ulna	1.96 ± 0.07	1.93 ± 0.06	1.18 ± 0.05	40	1.15 ± 0.03	41
		(p > 0.18)				

Results are reported as mean ± standard deviation.

Results from further testing on the tibial samples (Table 2) showed that demineralisation with 0.6 M and 1.2 M HCl also produced significant reductions in vBMD, of 25% and 30% respectively (both p < 0.001). The different preparation conditions for the tibial samples (R/O, PBS, fresh) did not generate significant differences in vBMD.

**Table 2:** Raw and adjusted pullout forces and vBMD for different preparation and demineralisation techniques.

	Density (gcm^−3^)	Mean cortical thickness (mm)	Pullout force (raw) (N)	Adjusted Pullout force for equivalent of 3.3 mm thick cortices (N)
Fresh Tibia	1.93 ± 0.08	3.37	920.7 ± 155.3	900.5 ± 103.7
Reverse Osmosis	1.85 ± 0.08	3.81	969.2 ± 231.2	839.7 ± 160.0
Phosphate Buffered Solution	1.87 ± 0.08	3.53	905.8 ± 190.6	847.1 ± 87.5
0.6 M HCl	1.44 ± 0.04	3.43	271.5 ± 175.0	260.7 ± 142.0
1.2 M HCl	1.35 ± 0.05	2.71	115.9 ± 29.6	142.4 ± 11.3
2.4 M HCl	1.19 ± 0.04	2.81	48.3 ± 12.4	56.7 ± 12.3
Dehydrated Tibia	1.66 ± 0.03	3.83	775.2 ± 250.5	667.9 ± 162.8
Reverse Osmosis Dehydrated	2.08 ± 0.04	3.74	595.9 ± 136.2	527.3 ± 124.7
Phosphate Buffered Solution Dehydrated	1.89 ± 0.11	4.24	1082.0 ± 294.9	842.1 ± 245.5
0.6 M HCl Dehydrated	1.58 ± 0.06	2.86	434.2 ± 178.7	502.0 ± 101.6
1.2 M HCl Dehydrated	1.35 ± 0.07	3.11	114.3 ± 27.3	121.2 ± 12.0
2.4 M HCl Dehydrated	1.25 ± 0.06	2.19	41.6 ± 18.8	61.5 ± 21.8

Dehydration of the samples produced varied results. There was a significant reduction in vBMD upon dehydrating the fresh tibia, 0.6 M and R/O samples (all p < 0.001), but not with 1.2 M, 2.4 M and PBS samples (Table 2). Combining dehydration with demineralisation did not reduce the vBMD further than either method (demineralisation or dehydration) alone (Table 3).

**Table 3:** Comparison of demineralised and dehydrated tibial volumetric bone mineral density (gcm^−3^) (percentage reduction compared to fresh tibia).

	No demineralisation	0.6 M HCl	1.2 M HCl	2.4 M HCl
No dehydration	1.93 ± 0.08	1.44^a^ ± 0.04 (25%)	1.35^a^ ± 0.05 (30%)	1.19^a^ ± 0.04 (38%)
Dehydrated	1.66^a^ ± 0.03 (14%)	1.58^a,b^ ± 0.06 (18%)	1.35^a^ ± 0.07 (30%)	1.25^a^ ± 0.06 (35%)

^a^Different from Fresh tibia (p < 0.001).

^b^Different from undehydrated sample (p < 0.001).

### Pullout testing

**Figure 6:**
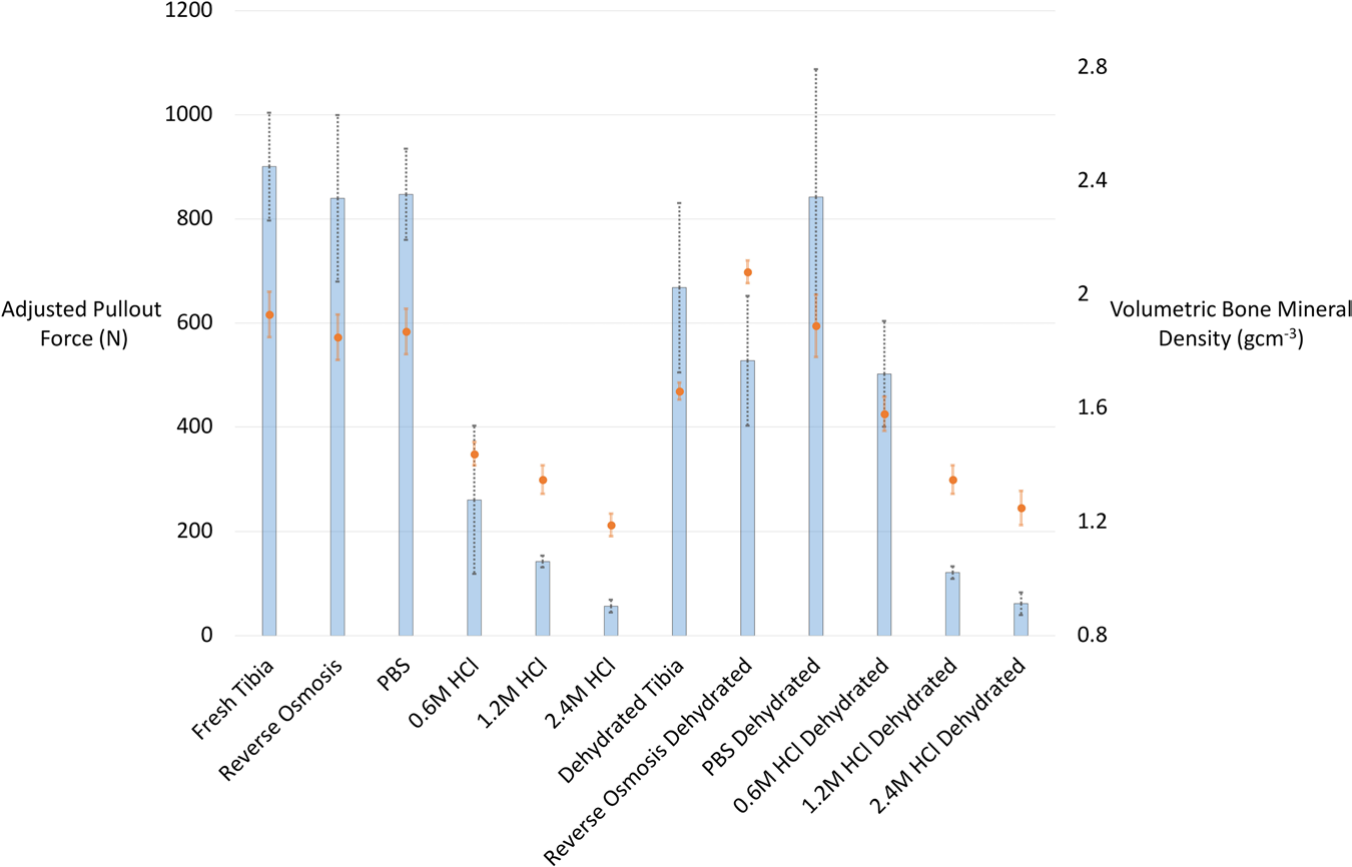
Adjusted pullout force and volumetric bone mineral densities for different tibial preparation methods (Bars represent adjusted pullout force (N); Dots represent volumetric bone mineral density (gcm^−^^3^)).

The mean cortical thickness was 3.3 ± 0.6 mm. Equations linearly relating cortical thickness and pullout strength were generated for each test condition, using:

$$\begin{array}{lrl}Adjusted\,pullout\,force & = & raw\,pullout\,force+((mean\,cortical\,thickness\\  &  & -test\,cortical\,thickness)\times (adjustment\,{coefficient}^{\ast }))\\ {}^{\ast }{Adjustment}\,{coefficient} & = & pullout\,force/cortical\,thickness\,(different\,for\,each\,test\,condition)\end{array}$$

where the adjustment coefficient ranged between 17 for 2.4 M HCl and 273 for fresh tibia. Pullout forces were highest with fresh, R/O and PBS samples, with no significant difference between these (Table 2). Demineralisation caused significant decreases in pullout force for all acid concentrations, with the following respective mean percentage reductions: 0.6 M: 71%, 1.2 M: 84% and 2.4 M: 94%. Similar reductions were seen with the dehydrated samples compared to fresh, except for the 0.6 M samples: dehydrated tibia: 26%, 0.6 M: 44%, 1.2 M: 87% and 2.4 M: 93% (all dehydrated samples) (Fig. 6).

## Discussion

Juvenile bovine long bones have bone densities and biomechanical properties that make them suitable for use in orthopaedic research. This study establishes, for the first time, this material as a novel, suitable model of normal and, following demineralisation, osteoporotic human bone that basic and applied research can utilise. The low variability demonstrated in bone density and pullout force, and its customisable potential, highlight the benefits over current models, with the added advantages from reduced ethical restrictions.

Comparable vBMD were found amongst all four types of juvenile bovine long bone (humerus, ulna, femur and tibia). All untreated samples’ vBMD are within the normal range of healthy human adult bone density (1.2–3.0 gcm^−3^)^3,4^ and very closely match the findings from one study (adult male, cortical bone density ranging for femora from 1.85–1.93 gcm^−3^ and for tibiae 1.83–1.96 gcm^−3^)^17^. This ensures that, relating to this characteristic alone, juvenile bovine bone closely resembles normal human bone whilst demonstrating very low variability within and between all long bone types examined; especially compared to the variability seen within and between some human samples^3,4^. This may in part due to the animals being reared in identical conditions to each other, ensuring variations from environmental factors remain minimised alongside similar baseline genetics. Additionally, several of these comparable animals will be slaughtered at the same age, providing an even more homogeneous sample set at the time of procurement.

The objective of demineralisation is to reduce the vBMD to levels similar to those present in the target population. Indeed, all demineralisation concentrations (0.6 M, 1.2 M and 2.4 M) reduced vBMD, generating a spectrum of changes in bone density compared to fresh tibiae, with reductions of 25%, 30% and 38% respectively. Other studies using broadly similar acid demineralising techniques generated decreases of 22% in areal BMD^8^ and 12% in vBMD^10^ using 0.6 M HCl, and vBMD reductions of 28% and 44% with 1.2 M and 2.4 M respectively^10^.

The reduction in pullout force seen with these demineralised models validates them biomechanically as they are in keeping with the loss of strength observed in osteoporotic bone. Additionally, these results are similar to previous research groups’ findings using axial bovine skeleton; for 0.6 M HCl, the 71% decrease in pullout force seen for the 25% reduction in vBMD compares to 59% reduction in pullout force following a 22% reduction in areal BMD^8^.

The models created mimic the reductions in vBMD needed to successfully recreate osteoporotic bone with the three demineralisation concentrations creating a variety of densities. However, when creating models, the target bone density should be considered comparatively to the target population mean rather than arbitrary values for diseased bone. The World Health Organisation defines osteoporosis as a bone density being 2.5 standard deviations below the mean^18^; thus establishing whether a bone density is osteoporotic is dependent on the population mean, which will vary between demographics. Further to this, more than 50% of ‘fragility fractures’ (fractures that occur from standing height or less) do not occur in osteoporotic bone, but in osteopenic bone (1 to 2.5 SD below the population mean)^19^. Therefore whilst the actual value of the vBMD is important, and quantitative definitions for osteopenia and osteoporosis are available^18^, it is the comparison between the normal population’s vBMD and the diseased model’s vBMD that is most important.

Demineralisation causes changes to bone properties by altering chemical composition and calcium content. These result in increased cortical porosity^10^, overall reduction in water content (though actually increased pore water content), loss of hardness, reduced compressive strength^20^, decreased material stiffness and decreased toughness^6,21^. In humans, cortical porosity increases with age and contributes to the detrimental properties seen in osteoporotic bone; it negatively correlates with bone strength^22^ and increases bone fragility^21^. Untreated juvenile bovine bone has been shown to be more porous than mature bovine bone^23^. Further to this, whilst the general microstructure of cortical bovine bone is thought to be preserved despite demineralisation^10^, the process does lead to increased cortical porosity, reduced vBMD and reduced collagen content; affecting both the quantity and quality of the bone^24^ as per the aim of an osteoporotic model. Whilst the cortical porosity was not directly measured during this study, it has been shown that vBMD can be used as a surrogate for it^25^, alongside differences in cortical porosity explaining 76% of the changes seen in ultimate tensile strength^21^. The methods employed (using juvenile bone and demineralisation) are likely to have changed the pore sizes in a manner representative of those seen in reduced density bone, given previous investigations of demineralisation^10^ and the reduction in tensile strength seen.

It is known that the total water content of bone and its toughness decrease with age^26,27^ and that reductions in water content lead to a reduced fracture resistance^28^. Dehydration of bone samples stiffens collagen and bone^29^, however it is unclear exactly what happens to water distribution with bone aging^29^. Given the complicated distribution of water in bone, our simple method of drying samples did not refine the models further; whilst changes in vBMD and pullout forces were seen between dried and non-dried samples, these were generally neither significant nor consistent.

It has been noted by other research groups that these demineralisation techniques do not fully remove collagen so may represent osteomalacia more than osteoporosis^8^. Additionally, other parameters are yet to be assessed to fully validate these models, such as evaluating mineral to matrix ratios, water content, pore size, bone microstructure, hardness and toughness. Degradation methods similar to ours have been used by other research groups and have been shown to affect bone in the desired way, such as increasing pore size. However, the lack of assessment of bone quality, beyond tensile testing, limits this study^30^. This may constrain the suitability of the model in mimicking all the conditions found in fracture fixations. Nevertheless, the reliability and low variability of its biomechanical properties, and its macroscopic dimensions, display the key aspects needed for experimental models to facilitate clinically relevant orthopaedic research. Further to this, our methods employed simple techniques for procurement, preparation and degradation, to produce significant reductions in vBMD. Whilst routinely available safety equipment is required during demineralisation, no other special equipment is needed for the storage and disposal of specimens given that they can be treated as part of the food chain. No changes in the solutions more frequently than 24 hours are needed as there would only be negligible changes in acid bath concentration during demineralisation. Additionally, treatment for more than 24 hours caused no further significant reduction in vBMD; confirming previous findings^31,32^. The strongest acid concentrations did significantly reduce the vBMD but in doing so macroscopically damaged the bone structure, creating very soft, malleable samples alongside reducing the cortical thickness from 3.3 mm (fresh tibia) to 2.8 mm (2.4 M HCl). These samples had very low maximum insertion torque levels (0.1 Nm) and very low pullout forces (~94% less than fresh samples). Given that the 0.6 M and 1.2 M solutions reduced vBMD without additional softening problems, we recommend using these concentrations for a reduced bone density model, though there may be further post demineralisation treatments that could be employed to stabilise the 2.4 M samples.

Many studies that use pullout force to biomechanically validate models do not explicitly specify their testing methods, especially whether they controlled for the insertion torque applied. By adjusting for other variables such as cortical thickness and controlling variables such as the drill insertion and axial pullout angles, pullout testing will have been less affected by confounding factors. The insertion torque value used, of 0.5 Nm, is within the range seen in human cortical bone^33^, and was found to be approximately 50% of the stripping torque for all specimens except for 1.2 M and 2.4 M samples. The pullout testing itself may not represent clinical screw failure methods accurately, as there is rarely a single catastrophic event in fixation failure. However, this method reduces confounders and is easily reproducible when assessing different preparation solutions, whilst being a testing method employed in many other studies.

Our model concentrates on cortical bone, both for simplicity and as cortical bone characteristics are far more significant in fracture mechanics and in dictating the fragility of bone; the trabecular bone contributes a trivial role to the biomechanical behaviours of bone (cancellous bone contributes <10% of bone strength^34^). Indeed, it has been shown that when cortices are >1.5 mm, the cortical thickness alone significantly influences pullout strength independent of the trabecular bone^35^. Further research into the validity of modelling longer bone sections plus research into the compressive strength and other biomechanical properties is warranted given the significant role this model could have in future advancements in biomechanics.

Ethical and financial constrains using juvenile bovine bone are minimal, especially compared to alternative animal and synthetic bone models. The cost for a single, standard-sized *in vitro* tibial model compared to one juvenile bovine bone are as follows: normal density foam sawbone x16, osteoporotic sawbone x69, 4th generation sawbone x185^36,37^ and cadaveric human tibia approximately x500^38^. Furthermore, these prices do not reflect the significant associated costs with storage, shipping, use and disposal of human specimens, or the costs associated with creating *in vivo* models. Factoring in demineralisation materials, one reduced vBMD juvenile bovine tibia was generated for less than $5.

This study provides, for the first time, quantitative assessment of juvenile bovine long bones, and the effects of demineralisation on them. The similarities seen amongst the different long bones tested demonstrate that their vBMD would make them suitable for tests mimicking human bone. Given the macroscopic dimensions of juvenile bovine tibiae, that they are inexpensive, readily available, not subject to ethical limitations, demonstrate low variability and can be demineralised to modify their bone density, they can be utilised as a model for biomechanical and fracture fixation testing of both normal and reduced density bone conditions.
